# Phenylalkanoid Glycosides (Non-Salicinoids) from Wood Chips of *Salix triandra* × *dasyclados* Hybrid Willow

**DOI:** 10.3390/molecules24061152

**Published:** 2019-03-23

**Authors:** Clarice Noleto-Dias, Yanqi Wu, Alice Bellisai, William Macalpine, Michael H. Beale, Jane L. Ward

**Affiliations:** Rothamsted Research, West Common, Harpenden, Hertfordshire AL5 2JQ, UK; clarice.noleto-dias@rothamsted.ac.uk (C.N.-D.); wuyanqi@zjut.edu.cn (Y.W.); alice.bellisai@rothamsted.ac.uk (A.B.); william.macalpine@rothamsted.ac.uk (W.M.); mike.beale@rothamsted.ac.uk (M.H.B.)

**Keywords:** phenylalkanoids, phenolic glycosides, *Salix triandra*, *Salix dasyclados*, chavicol, rosarin

## Abstract

*Salix triandra* (almond leaved willow) is an established crop, grown in coppicing regimes for basket-making materials. It is known as a source of non-salicinoid phenolic glycosides, such as triandrin and salidroside. A spontaneous natural hybrid of *S. triandra* and *S. dasyclados* was subjected to metabolite profiling by high resolution LC-MS, and 22 phenolic glycosides, including 18 that are new to the Salicaceae, were identified. Structures were determined by HPLC isolation and NMR methods. The hybridisation process has introduced novel chemistry into the Salix phenolic glycoside palette, in particular, the ability to generate disaccharide conjugates where the glycosyl group is further extended by a range of sugars, including apiose, rhamnose, xylose, and arabinose. Also of note is the appearance of chavicol derivatives, also not previously seen in *Salix* spp. The work demonstrates the plasticity of the phenolic glycoside biosynthetic pathway, and the potential to improve established crops such as *S. triandra* and *S. dasyclados*, via high-value metabolites, for both basketry and bioenergy markets.

## 1. Introduction

The Salicaceae family is a distinct taxon of perennial woody, dioecious shrubs and trees that can primarily be divided into two genera—willows (*Salix* spp.) and poplars (*Populus* spp.). Both groups have a rich secondary chemistry based on phenolic glycosides. The genus *Salix* is the largest and includes over 400 species that are variable in growth form, from large trees to small shrubs, and are distributed over a wide range of habitats [1]. Willow bark preparations have been used for the treatment of fever and pain since ancient times [2], and these bioactivities have mostly been related to their constitutive salicinoids, which are defined as derivatives of salicyl alcohol with β-d-glucopyranose moieties (e.g., salicin and salicortin) [3]. Although salicinoids are the most commonly studied class of secondary metabolites in the Salicaceae, other phenolic glycosides, as well as lignans, flavonoids, and terpenes, have been characterised [4]. Considering the variety of compounds described in willows, the beneficial effects of herbal products may not be ascribed only to salicinoids. Hydroxycinnamic acid and benzoic acid derivatives, for example, have potential antioxidant, antimicrobial and anticancer activities. These and other mono- and 1,4-*di*-substituted benzenoids may add value to short rotation coppice (SRC) willow crops [5], not only for such biomedical uses, but also, if present in a sufficient quantity, as feedstocks for aromatic bio-refining streams.

One of the best known species of willow that is not rich in salicinoids is *S. triandra* L. (almond-leaved willow) [6,7]. This straight-stemmed shrubby species is commonly grown in plantations that are coppiced annually, and is used for basketry, and varieties such as “Black Maul”, which can grow 2–3 m/year, are major contributors to this industry. As a part of our ongoing research towards adding value to willow crops, we are surveying the *S. triandra* species contained in the 1500+ U.K. National Willow Collection (NWC), maintained as a short-rotation coppice plantation at Rothamsted Research, Harpenden, United Kingdom. In this paper, we report on the secondary metabolite profile of wood-chips of an unusual *Salix triandra* × *dasyclados* hybrid (*S.* × *schaburovii* I.Beljaeva) (NWC1283). This naturally occurring hybrid originates from the flood plain of the Kama River, Perm, Russia, and came to the NWC in 2004 from the Botanical Gardens of the Urals. The study builds on our previous work [8,9] in the analysis of secondary metabolites in willow, particularly in the polar metabolites obtained from aqueous alcohol extractions [10].

## 2. Results and Discussion

**Figure 1:**
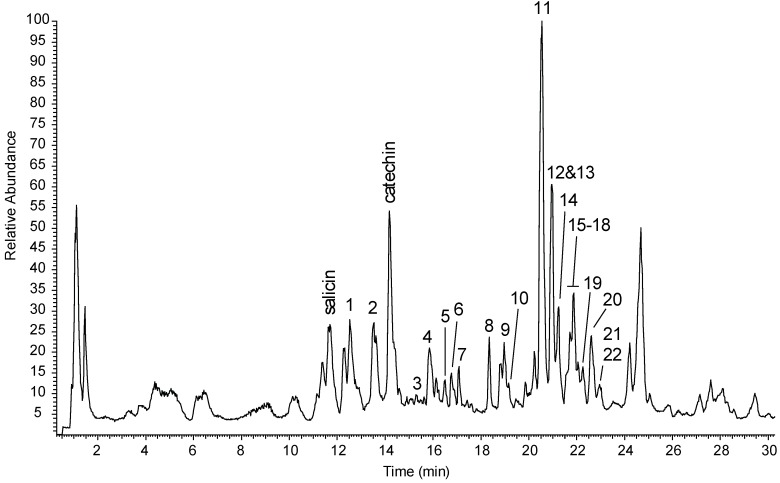
Total ion chromatogram (negative ion mode) extract generated from the *Salix triandra* × *dasyclados* hybrid (NWC1283) following extraction in water:ethanol (4:1).

Freeze-dried wood chips derived from the multiple whole stems of *S. triandra* × *dasyclados* (NWC 1283) grown in the field and harvested at the dormant stage (January), were extracted with aqueous ethanol and were analysed using UHPLC–HRMS. The total ion chromatogram (Figure 1) showed that this extract is a complex mixture of compounds, and, unlike most willow extracts, is not dominated by salicinoids, that is, salicin is a relatively minor peak, while salicortin, normally appearing at 20.5 min, is now totally obscured by more abundant peaks.

Peaks 1, 2, 11 and 14 were identified as picein, salidroside, rosarin and rosavin, respectively, in comparison with the ^1^H-NMR and LC–MS data (Table 1) of the authentic standards run under identical conditions. Picein (**1**) has been detected in different parts of the *Salix* species, such as in the leaves of *S. matsudana* [11] and in the bark of *S. purpurea* [12]. Salidroside (**2**) is also a major component in the leaves of *S. triandra* and of its hybrids with *S. viminalis* and *S. purpurea* [13].

**Figure 2:**
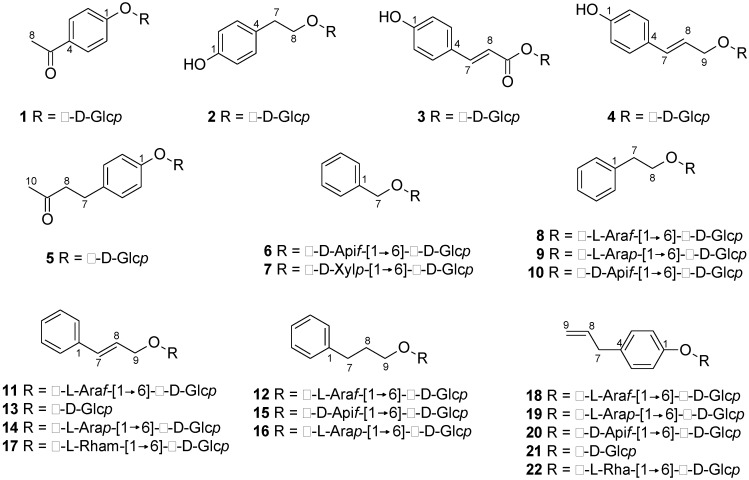
Chemical structures of phenylalkanoid glycosides identified in the hydroalcoholic extract of *S. triandra* × *dasyclados* hybrid (NWC1283) chipped biomass.

In order to identify further peaks, a portion of the extract was fractionated by semi-preparative HPLC via repeated injections. After the solvent removal, these fractions were analysed by NMR. In total, 22 phenylalkanoid glycosides were identified on the basis of the obtained HRMS and/or NMR data, compared with those found in the literature. Table 1 includes the UHPLC–HRMS data and the method of identification for these compounds, while their chemical structures can be found in Figure 2. Nineteen of the identified compounds are described for the first time in a *Salix* genotype, and one of them (**16**) is a novel molecule.

Peak 3 at 15.2 min with [M − H]^−^ at *m*/*z* 325.0930 had the molecular formula of C_15_H_18_O_8_, and was identified as *p*-coumaroyl-β-d-glucopyranoside based on its ^1^H-NMR peaks (Table 2) [14]. The molecule contained a pair of aromatic doublets, each with an 8.6 Hz coupling, at δ 6.96 and 7.60, indicating a para substituted molecule. A β-glucoside moiety was confirmed via inspection of the anomeric signal, which appeared as a doublet at δ 5.65 and had an 8 Hz coupling. Peak 4 at 15.8 min corresponded to the known compound triandrin, in which *m*/*z* 357.1187 arises from its formate adduct and 311.1133 from its molecular ion [15]. Triandrin (**4**) has been recently found in the leaves and twigs of *S. reticulata* [16], but is better known in *S. triandra* [6,7], a parent of NWC1283. Compound **5** eluted at 16.3 min and showed *m*/*z* 371.1347 [M − H + HCOOH]^−^. A comparison of its NMR data to those of a compound isolated from the roots of *Rheum officinale* Baill. (Polygonaceae) confirmed it to be *p*-hydroxybenzylacetone-β-d-glucopyranoside [17] (Table S1).

Compound **21** eluted at 22.9 min with *m*/*z* at 341.1243, corresponding to the formate adduct of the molecular formula C_15_H_20_O_6_. The ^1^H-NMR spectrum of this compound presented aromatic (δ_H_ 7.07 and 7.23 ppm, *d*, *J* = 8.7 Hz) and anomeric (δ_H_ 5.04 ppm, *d*, *J* = 7.7 Hz) signals (Table 2) similar to those of compound **5**. This indicates that this molecule contains a *para*-disubstituted aromatic ring, and that the glucose moiety is *O*-linked to this ring. In addition to this, signals of an allylic side chain (terminal olefinic proton at δ 5.08 ppm; multiplet at δ 6.01 ppm and doublet at δ 3.35 ppm) were also detected and the structure of compound **21** was confirmed to be chavicol-β-d-glucopyranoside, a compound previously isolated from *Cedronella canariensis* (L.) Webb and Berth. (Lamiaceae) [18] and *Alpinia officinarum* Hance (Zingiberaceae) [19].

A further four peaks can be assigned to chavicol with a diglycosidic chain. Compounds **18** and **20** were isolated, and the MS/MS data of both compounds showed ions at *m*/*z* 133, corresponding to the loss of a hexose–pentose fragment (*m*/*z* 295). In compound **18**, two sugar moieties were confirmed from the ^1^H and ^13^C-NMR data of the anomeric protons (δ_H_/δ_C_ 5.04/103.5 and 5.00/111.2) (Table 3 and Table S2). A coupling constant of 7.7 Hz for the anomeric signal at δ 5.04 suggested a β-glucoside linkage to the chavicol, whilst the smaller (1.3 Hz) coupling of the anomeric signal at δ 5.00 indicated an α-linkage of the pentose entity. The ^1^H-NMR signals agreed well with the published literature data for chavicol-α-l-arabinofuranosyl-(1→6)-β-d-glucopyranoside [20]. The furanosyl form of the arabinose moiety was confirmed by the downfield shift of the 1″-anomeric proton, appearing at δ 5.00, and the ^13^C resonance at δ 111.2 [21]. Chavicol-β-d-apiofuranosyl-(1→6)-β-d-glucopyranoside was the structure assigned to compound **20**. This was confirmed by the coalescence of the 5″-H_2_ signals to a singlet, appearing at δ 3.60, and characteristic of the apiose moiety. In addition, a quarternary signal was evident from the ^13^C-NMR data at δ 82.3 (Table 3 and Table S2). Both compound **18** and **20** have previously been found in *Betula papyrifera* Marsh. (Betulaceae) [20], and the NMR data was consistent for both compounds. Detailed ^1^H- and ^13^C-NMR spectroscopic data, including 2D correlations, can be found in Table S2. Peak 19, with the same molecular formula (C_20_H_28_O_10_), was putatively annotated as chavicol-α-l-arabinopyranosyl-(1→6)-β-d-glucopyranoside, because of its proximity to **18** in the chromatographic run (less than 0.2 min later), and that **19** is an impurity in the ^1^H-NMR spectrum of 18, being detected by the anomeric proton of the arabinopyranosyl moiety (δ_H_ 4.46 ppm, *d*, *J* = 8.0 Hz). The fourth compound eluted at 23.0 min with *m*/*z* at 441.1766, and was identified as chavicol-rutinoside (compound **22**) [19].

Monosubstituted aromatic glycosides were also identified in this study. Two compounds with molecular ions at *m*/*z* 401 (compounds **6** and **7**) and three at *m*/*z* 415 (compounds **8**–**10**) had their molecular formulas calculated for C_18_H_26_O_10_ and C_19_H_28_O_10_, respectively, based on accurate masses. These five compounds were purified for structural characterization, and the NMR data identified them as benzyl-β-d-apiofuranosyl-(1→6)-β-d-glucopyranoside (**6**), benzyl-β-d-xylopyranosyl-(1→6)-β-d-glucoside (**7**), previously described in *Jasminum sambac* Ait. (Oleaceae) [22], 2-phenylethyl-α-l-arabinofuranosyl-(1→6)-β-d-glucopyranoside (compound **8**), 2-Phenylethyl-α-l-arabinopyranosyl-β-d-glucopyranoside (compound **9**) and 2-Phenylethyl-β-d-apiofuranosyl-β-d-glucopyranoside (compound **10**). Their ^1^H-NMR chemical shift data is presented in Table 4. Compounds **6**, **7**, **9** and **10** have been detected in the aerial parts of *Hylomecon vernalis* Maxim. (Papaveraceae) [23].

Peaks 11, 13, 14 and 17 correspond to the cinnamyl alcohol derivatives rosarin, rosin, rosavin and cinnamrutinose A, respectively (Table 2 and Table 4). The first three compounds, together with salidroside (**2**), are commonly found in *Rhodiola rosea* L. (Crassulaceae) [24], and the NMR data was in agreement with the published values. Cinnamrutinose A (**17**), on the other hand, has been isolated from a more taxonomically similar species, *Populus tremula* (Salicaceae) [25].

Compound (**12**) had [M − H]^−^ at *m*/*z* 429.1767, corresponding to the molecular formula C_20_H_30_O_10_. The NMR data of this isolated compound was comparable to dihydrocinnamyl alcohol-α-l-arabinofuranosyl-(1→6)-β-d-glucopyranoside) (dihydro-rosarin), formerly described in *Juniperus communis* var. *depressa* [26]. Compound (**16**) showed the same molecular formula, however, the NMR data indicated a different sugar moiety (Table 5). The 1D ^1^H-NMR spectrum revealed the presence of a monosubstituted benzene ring and a hydroxypropyl group, as well as two sugars, a β-glucopyranose (anomeric proton at δ_H_ 4.42/δ_C_ 105.9) and a α-arabinopyranose (anomeric proton at δ_H_ 4.53/δ_C_ 104.4). Long range correlations observed between H-7/C-1 (δ_H_ 2.71/δ_C_ 145.3), H-1/C-9 (δ_H_ 4.42/δ_C_ 72.8) in the heteronuclear multiple bond correlation (HMBC) spectrum indicated how each moiety was connected in the molecule. Thus, the structure of 16 was determined to be dihydrocinnamyl alcohol-α-l-arabinopyranosyl-(1→6)-β-d-glucopyranoside (dihydro-rosavin), and this compound has not previously been reported in the literature.

Although compound **15** could not be purified, the UHPLC–MS chromatogram of the fraction that contains **16** indicates that a small proportion of 15 can be detected in it (two peaks not well resolved, data not shown). In order to find more evidence of the structure of **15**, a careful analysis of the 1D ^1^H and 2D ^1^H–^13^C-HSQC and HMBC-NMR spectra of 16 was performed. Some minor peaks corresponding to a β-apiofuranosyl moiety were observed, such as an anomeric at δ_H_ 5.07 ppm (d, *J* = 3.2 Hz)/δ_C_ 111.9 ppm (CH-1″); δ_H_ 3.96 ppm (d, *J* = 3.2 Hz)/δ_C_ 79.6 ppm (CH-2″); δ_C_ 82.3 ppm (C-3″); δ_H_ 4.00 ppm (d, *J* = 10.1 Hz), 3.85 ppm (d, *J* = 10.1 Hz)/δ_C_ 76.7 ppm (CH_2_-4″); and δ_H_ 3.62 ppm (s)/δ_C_ 66.5 ppm (CH_2_-5″). The relative integration of these peaks to the corresponding signals of 16 was 1:3. Together with this data, the fact that 15 presented the same molecular formula and mass fragments as **16**, and the proximity of them in the UHPLC run (less than 0.2 min), we can suggest that **15** is dihydrocinnamyl alcohol-β-apiofuranosyl-(1→6)-β-glucopyranoside.

In summary, the hybrid of *S. triandra* and *S. dasyclados* (NWC1283) contains a much larger array of chemistry, particularly of phenylalkanoid disaccharides, than has previously been reported in either of the parental species. Apart from salidroside (**2**), which was the only phenyethanoid glycoside previously known in the Salicaceae [27], many of the compounds reported have not been reported from willow before. Of particular note is the appearance of the rosavin, rosarin and chavicol analogues, and the propensity of these and others to contain disaccharide groups, where the “normal *Salix*” glycosyl moiety is further substituted at C-6 by arabinose, apiose, xylose or rhamnose. This second glycosidic substitution can also consist of either the pyranosyl form or of the furanosyl form, and evidence of both forms is present for several of the compounds isolated. As evidenced in this study, such compounds can be isolated directly from the chipped biomass with relatively straightforward extraction techniques. In this study, extraction was achieved using 20% aqueous ethanol, a solvent system that is cheap and therefore applicable to larger scale extractions. Similarly, heating steps were not required to release the compounds reported, and extraction at room temperature preserved the integrity of the compounds. This work also demonstrates the potential of plant breeding to introduce traits that result in new chemistry, potential bioactivities and provide the opportunity to add value to basketry and bioenergy crops.

## 3. Materials and Methods

### 3.1. General Experimental Procedures

The ^1^H-1D and ^1^H–^1^H and ^1^H–^13^C 2D-NMR spectra of each compound were acquired, using a 5 mm triple resonance (TCI) cryoprobe, on a Bruker Avance 600 MHz NMR spectrometer (Bruker Biospin, Germany), operating at 600.05 MHz for ^1^H-NMR and 150.9 MHz for the ^13^C-NMR spectra. Typical 1-dimensional ^1^H spectra were obtained with an acquisition time of 4.6 s, a sweep width of 7142.9 Hz and 65,536 data points. A total of 16 scans were recorded using the zgpr pulse sequence with a 90° angle. A relaxation delay of 5 s was used to suppress the residual HOD signal. The spectra were transformed using an exponential window with a line broadening of 0.5 Hz. ^1^H–^1^H correlation spectroscopy (COSY) were run using the pulse sequence cosyprqf for 3 h, and the frequency was 600.05 MHz in both dimensions. The acquisition times were 0.1434 and 0.0896 s, and the sweep widths were 7142.9 Hz. There were 1024 data points collected in each dimension using 32 transients. ^1^H–^13^C heteronuclear single quantum coherence (HSQC) spectra were performed using the pulse sequence hsqcetgpsi2 for 10 h, at 600.05 and 150.9 MHz frequencies, with acquisition times of 0.1433 and 0.00212 s. The data were acquired using sweep widths of 7142.9 and 30,120.5 Hz. There were 2048 and 1024 data points collected using 128 transients. The ^1^H–^13^C heteronuclear multiple bond correlation (HMBC) spectra were obtained using the pulse sequence hmbcgpndqf for 22 h. The acquisition parameters were the same as stated for the HSQC data collection. For comparability with previous work [10], all of the spectra were collected at 300 °K in D_2_O:CD_3_OD (8:2), and chemical shifts are given in δ, relative to TSP-*d*_4_ ((trimethylsilyl) propionic acid, 0.01 % *w*/*v*) added as a chemical shift reference standard. The compound concentration was typically 1 mg/mL. Phasing and baseline correction were carried out within TOPSPIN v. 2.1 (Bruker Biospin, Germany). Structural assignments of carbohydrate moieties were made with reference to the authentic standards and the use of characteristic chemical shift data [21].

UHPLC–MS were recorded on an LTQ-Orbitrap Elite mass spectrometer (Thermo Fisher, Bremen, Germany) coupled to a Dionex UltiMate 3000 RS UHPLC system, equipped with a DAD-3000 photodiode array detector. Separation was carried out in reverse-phase using Hypersil GOLD™ column (1.9 μm, 30 × 2.1 mm i.d. Thermo Fisher Scientific, Germany), which was maintained at 35 °C. The solvent system consisted of water/0.1% formic acid (A) and acetonitrile/0.1% formic acid (B), both Optima™ grade (Thermo Fisher Scientific, Germany). The injection volume was 10 μL and separation was carried out for 40 min with a flow rate of 0.3 mL/min under the following gradient: 0–5 min, 0% B; 5–27 min, 31.6% B; 27–34 min, 45% B and 34–37.5 min, 75% B. The mass spectra were collected in negative ion mode using a heated electrospray source (Thermo Fisher Scientific, Germany). The resolution was 120,000 over *m*/*z* 50–1500, and the source voltage, sheath gas, auxiliary gas, sweep gas and capillary temperature were set to 2.5 kV, 35 (arbitrary units), 10 (arbitrary units), 0.0 (arbitrary units) and 350 °C, respectively. Automatic MS–MS was performed on the four most abundant ions using an isolation width of *m*/*z* 2. The ions were fragmented with a normalised collision energy of 65 and an activation time of 0.1 ms, using high-energy C-trap dissociation. The data were collected and inspected using Xcalibur v. 2.2 (Thermo Fisher Scientific, Germany).

The compounds were isolated by repeated injection into an HPLC system (Dionex UltiMate 3000, Thermo Fisher Scientific) equipped with an Ascentis C-18 column (5 μm, 5 × 250 mm i.d., Sigma-Aldrich, Gillingham, UK). The column was maintained at 35 °C and chromatographic separation was performed using a constant flow rate of 1 mL/min. The mobile phases were water (A) and acetonitrile (B), both containing 0.1% formic acid. To achieve separation, the gradient used was as follows: 0–2 min, 5% B; 2–5 min, 12% B, 5–10 min, 12% B, 10–60 min and 40% B. The peaks were detected using UV wavelengths of 210 to 360 nm, and fractions corresponding to the target compounds were collected into glass tubes. Twelve injections (100 μL each) were performed and the fractions from repeated runs were combined and the solvent was evaporated using a Speedvac concentrator (Genevac, Suffolk, UK).

### 3.2. Plant Material and Metabolite Extraction

NWC1283 is one of 16 genotypes that makes up CS/782. CS/782 was planted using winter dormant 20 cm cutting in May 2017 at Rothamsted Research (Hertfordshire, UK) (51°48′ N, 0°21′ W). The previous crop was winter wheat. The soil type within the field is silty clay loam with flints over clay. Planting and agronomy followed the conventional SRC best practice. Willows were planted as cuttings using the typical twin-row design at a planting density of 16,667 plants ha^−1^, establishment year growth received no fertilisers and two pre-emergence herbicides were applied within 10 days of planting Pendimethalin (Stomp at 3.3 l/ha) and Isoxaben (Flexidor at 1.0 l/ha). The genotypes were planted in unreplicated plots each containing 80 plants (two twin rows of 40).

The winter dormant above ground biomass was harvested in January 2018. The stems were cut 5 cm above the ground, and were immediately chipped in a Petrol Shredder 100 mm (4″). The material was stored at −80 °C and then freeze-dried. A voucher specimen has been retained and is available on request.

The chips (60 g) were extracted at room temperature, by soaking with 400 mL water:ethanol (4:1) for 16 h. Aliquots were taken for initial metabolite profiling by ^1^H-NMR and UHPLC-MS and for compound isolation by HPLC.

## Figures and Tables

**Table 1:** Phenylalkanoid glycosides identified in the hydroalcoholic extract of *Salix triandra* × *dasyclados* (NWC1283) wood chips.

No.	[M − H]^−^ (*m*/*z*)	t_R_ (min)	Formula	Δ (ppm)	MS/MS Ions (*m*/*z*)	Compound
**1**	297.0981	12.6	C_14_H_18_O_7_	+0.55	**135**, 181	Picein ^a^
**2**	299.1136	13.4	C_14_H_20_O_7_	−0.03	119, **137**, 179	Salidroside ^a^
**3**	325.0930	15.2	C_15_H_18_O_8_	+0.29	89, 119, 145, **163**, 193	*p*-Coumaroyl-β-d-glc*p*^b^
**4**	311.1133	15.8	C_15_H_20_O_7_	−1.11	**149**, 161	Triandrin ^b^
**5**	325.1289	16.3	C_16_H_22_O_7_	+0.47	**163**	*p*-Hydroxybenzylacetone-β-d-glc*p*^b^
**6**	401.1453	16.7	C_18_H_26_O_10_	+0.01	161, **269**	Benzyl-β-d-api*f*-(1→6)-β-d-glc*p* ^b^
**7**	401.1454	17.1	C_18_H_26_O_10_	+0.31	161, **269**	Benzyl-β-d-xyl*p*-(1→6)-β-d-glc*p* ^b^
**8**	415.1611	18.3	C_19_H_28_O_10_	+0.31	179, **191**, 283	2-phenylethyl-α-l-ara*f*-(1→6)-β-d-glc*p*
**9**	415.1612	18.9	C_19_H_28_O_10_	+0.46	**149**, 179, 191, 283	2-Phenylethyl-α-l-ara*p*-(1→6)-β-d-glc*p* ^b^
**10**	415.1610	19.0	C_19_H_28_O_10_	+0.07	89, **149**, 191, 283	2-Phenylethyl-β-d-api*f*-(1→6)-β-d-glc*p* ^b^
**11**	427.1611	20.5	C_20_H_28_O_10_	+0.24	125, 133, 149, **161**, 191, 293	Rosarin ^a^
**12**	429.1768	20.9	C_20_H_30_O_10_	+0.32	101, 131, 161, **297**	Dihydrorosarin ^b^
**13**	341.1242 *	21.0	C_15_H_20_O_6_	+0.04	**133**, 161	Rosin ^b^
**14**	427.1610	21.2	C_20_H_28_O_10_	+0.09	125, 133, 149, **161**, 191, 233, 293	Rosavin ^a^
**15**	429.1766	21.5	C_20_H_30_O_10_	+0.03	101, 131, 161, **297**	Phenylpropanol-β-d-api*f*-(1→6)-β-d-glc*p* ^c^
**16**	429.1767	21.6	C_20_H_30_O_10_	+0.11	101, 131, 161, **297**	Dihydrorosavin ^b^
**17**	441.1768	21.8	C_21_H_30_O_10_	+0.52	101, **125**, 163, 247, 307	Cinnamrutinose A ^b^
**18**	427.1611	21.9	C_20_H_28_O_10_	+0.38	89, 125, **133**, 191, 233, 293	Chavicol-α-l-ara*f*-(1→6)-β-d-glc*p* ^b^
**19**	427.1610	22.1	C_20_H_28_O_10_	+0.02	89, 125, 133, **149**, 191, 233, 293	Chavicol-α-l-ara*p*-(1→6)-β-d-glc*p* ^c^
**20**	427.1610	22.6	C_20_H_28_O_10_	+0.09	89, 125, 133, 149, 191, 233, **293**	Chavicol-β-d-api*f*-(1→6)-β-d-glc*p* ^b^
**21**	341.1243 *	22.9	C_15_H_20_O_6_	+0.31	**133**, 161	Chavicol-glucoside ^b^
**22**	441.1766	23.0	C_21_H_30_O_10_	−0.03	101, **125**, 163, 247, 307	Chavicol-rutinoside ^b^

* corresponds to the formate adduct. ^a^ Identification based on comparison with standards. ^b^ Identification based on isolation of the compounds and NMR. ^c^ Tentative identification based on the detection of some of its NMR and LC-HRMS signals as impurities of other compounds. Abbreviations: glc*p*—glucopyranoside; api*f*—apiofuranosyl; xyl*p*—xylopyranosyl; ara*f*—arabinofuranosyl; ara*p*—arabinopyranosyl. Numbers in bold represent the base peak.

**Table 2:** ^1^H-NMR data of monosaccharides found in *S. triandra* × *dasyclados* hybrid (NWC1283) wood chip extracts.

Position	(1)	(2)	(3)	(4)	(5)	(13)	(21)
1	−	−	−	−	−	−	−
2	7.22 *d* (8.9)	6.84 *d* (8.6)	6.93 *d* (8.6)	6.88 *d* (8.6)	7.07 *d* (8.6)	7.52 *d* (7.4)	7.07 d (8.7)
3	8.03 *d* (8.9)	7.21 *d* (8.6)	7.60 *d* (8.6)	7.41 *d* (8.6)	7.23 *d* (8.6)	7.41 *d* (7.6)	7.23 *d* (8.7)
4	−	−	−	−	−	7.34 *d* (7.5)	−
5	8.03 *d* (8.9)	7.21 *d* (8.6)	7.60 *d* (8.6)	7.41 *d* (8.6)	7.23 *d* (8.6)	7.41 *d* (7.6)	7.23 *d* (8.7)
6	7.22 *d* (8.9)	6.84 *d* (8.6)	6.93 *d* (8.6)	6.88 *d* (8.6)	7.07 *d* (8.6)	7.52 *d* (7.4)	7.07 *d* (8.7)
7	−	2.88 *t* (7.1)	7.84 *d* (15.9)	3.68 *d* (15.9)	2.86 *m*	6.77 *d* (16.3)	3.36 *d* (6.8)
8	2.64 *s*	4.08 dt (7.1, 10.1)	6.48 *d* (15.9)	6.26 *dt* (15.9, 6.6)	2.88 *m*	6.42 *m*	6.01 *ddt* (17, 10.1, 6.8)
		3.85 dt (7.3, 10.1)					
9	−	−	−	4.51 *ddd* (1.2, 6.2, 12.4)	−	4.55 *m*, 4.41 *m*	5.08 *d* (17.0)
				4.38 *ddd* (1.0, 7.0, 12.4)			
10	−	−	−	−	2.19 s	−	−
1′	5.23 *d* (7.5)	4.44 *d* (8.0)	5.65 *d* (8.0)	4.52 *d* (8.0)	5.05 *d* (7.7)	4.53 *d* (8.0)	5.04 *d* (7.7)
2′	3.60–3.62 *m*	3.23 *dd* (8.0, 9.3)	3.62–3.46 *m*	3.29 *dd* (8.0, 9.3)	3.53 *d* (7.8)	3.49–3.39 *m*	3.59–3.41 *m*
3′	3.60–3.62 *m*	3.45 *d* (9.0)	3.62–3.46 *m*	3.47 *d* (8.9)	3.58 *d* (9.0)	3.49–3.39 *m*	3.59–3.41 *m*
4′	3.52 *d* (9.7)	3.39 *d* (8.9)	3.62–3.46 *m*	3.39 *d* (9.6)	3.47 *d* (9.0)	3.49–3.39 *m*	3.59–3.41 *m*
5′	3.67 *ddd* (2.2, 5.7, 9.8)	3.40 *ddd* (2.2, 9.8, 6.7)	3.62–3.46 *m*	3.41 *ddd* (2.1, 5.7, 9.8)	3.43 *m*	3.49–3.39 *m*	3.59–3.41 *m*
6′	3.94 *dd* (2.2, 12.5)	3.89 *dd* (2.2, 12.3)	3.79 *dd* (2.0, 12.0)	3.90 *dd* (2.1, 12.3)	3.92 *dd* (2.1, 12.4)	3.91 *dd* (2.1, 12.3)	3.90 *dd* (2.3, 12.4)
	3.77 *dd* (5.7, 12.5)	3.70 *dd* (5.7, 12.3)	3.71 *dd* (5.2, 12.0)	3.72 *dd* (5.8, 12.3)	3.73 *dd* (5.8, 12.4)	3.72 *dd* (6.0, 12.2)	3.71 *dd* (5.6, 12.2)

Data collected in 80:20 D_2_O:CD_3_OD (4:1). Spectra were referenced to TSP-*d*_4_ ((trimethylsilyl) propionic acid, 0.01% *w*/*v*) at δ 0.00. Coupling constants in Hz are given in parentheses. Abbreviations: *s—*singlet; *d*—doublet; *t*—triplet; *dd*—doublet of doublets; *dt*—doublet of triplets; *ddt*—double doublet of triplets; *ddd*—doublet of double doublets; *m*—multiplet.

**Table 3:** ^1^H-NMR data of cinnamyl and chavicol disaccharides found in *S. triandra* × *dasyclados* hybrid (NWC1283) wood chip extracts.

Position	(17)	(18)	(20)	(22)
2	7.50 *d* (7.3)	7.07 *d* (8.7)	7.07 *d* (8.6)	7.07 *d* (8.6)
3	7.40 *d* (7.6)	7.22 *d* (8.7)	7.23 *d* (8.6)	7.23 *d* (8.6)
4	7.33 *d* (7.4)	−	−	−
5	7.40 *d* (7.6)	7.22 *d* (8.7)	7.23 *d* (8.6)	7.23 *d* (8.6)
6	7.50 *d* (7.3)	7.07 *d* (8.7)	7.07 *d* (8.6)	7.07 *d* (8.6)
7	6.74 *d* (16.0)	3.36 *d* (6.7)	3.36 *d* (6.9)	3.36 *d* (6.9)
8	6.39 *dt* (6.5, 16.0)	6.01 *ddt* (16.9, 10.1, 6.7).	6.01 *ddt* (16.9, 10.1, 6.7)	6.01 *ddt* (16.9, 10.1, 6.7)
9	4.50 *ddd* (12.7, 6.0, 1.2)	5.07 *m*	5.08 *m*	5.08 *m*
	4.4 *ddd* (1.1, 6.8, 12.7)			
1′	4.51 *d* (8.0)	5.04 *d* (7.7)	5.03 *d* (7.6)	5.03 *d* (7.6)
2′	3.46 *d* (9.1)	3.54 *d* (7.7)	3.53 *d* (7.6)	3.55–3.40 *m*
3′	3.45 *d* (9.1)	3.50 *d* (9.1)	3.56 *d* (8.9)	3.55–3.40 *m*
4′	3.41 *dd* (1.8, 9.6)	3.57 *d* (9.0)	3.48 *d* (9.2)	3.55–3.40 *m*
5′	3.53 *ddd* (1.8, 5.9, 9.6)	3.73 *m*	3.70 *m*	3.55–3.40 *m*
6′	3.97 *dd* (1.8, 11.6) 3.68 *dd* (5.9, 11.6)	4.04 *dd* (1.3, 11.1) 3.70 *m*	4.02 *dd* (2.3, 11.4) 3.72 *m*	4.12 *dd* (1.7, 11.9) 3.74 *m*
1″	4.52 *d* (1.7)	5.00 *d* (1.3)	5.05 *d* (3.1)	5.07 *d* (1.8)
2″	3.94 *dd* (1.8, 3.4)	4.06 *dd* (1.5, 3.3)	3.94 *d* (3.1)	4.09 *dd* (1.6, 3.3)
3″	3.77 *dd* (3.4, 9.7)	3.89 *dd* (3.3, 5.9)	−	3.75 *m*
4″	3.42 *d* (9.6)	4.00 td (3.3, 5.9)	3.83 *d* (10.1) 3.99 *d* (10.1)	3.40 *m*
5″	3.72 *dd* (6.3, 9.6)	3.74 *dd* (3.3, 12.3) 3.64 *dd* (5.7, 12.3)	3.60 *s*	3.72 *dd* (6.2, 9.3)
6″	1.27 *d* (6.3)	−	−	1.19 *d* (6.2)

Data collected in 80:20 D_2_O:CD_3_OD (4:1). Spectra were referenced to TSP-*d*_4_ at δ 0.00. Coupling constants in Hz are given in parentheses Abbreviations: *s*—singlet; *d*—doublet; *dd*—doublet of doublets; *ddt*—doublet of double triplets; *ddd*—doublet of double doublets; *m*—multiplet.

**Table 4:** ^1^H-NMR data of benzyl disaccharides found in *S. triandra* × *dasyclados* hybrid (NWC1283) wood chip extracts.

Position	(6)	(7)	(8)	(9)	(10)
2	7.47 *dd* (1.6, 8.2)	7.46 *m*	7.40–7.37 *m*	7.39–7.36 *m*	7.39–7.36 *m*
3	7.44 *dd* (7.1, 1.6)	7.44 *dd* (1.5, 7.6)	7.37–7.33 *m*	7.36–7.34 *m*	7.36–7.34 *m*
4	7.41 *d* (7.0)	7.41 *dd* (1.6, 8.7)	7.29 *t* (7.3)	7.29 *t* (7.0)	7.29 *t* (7.0)
5	7.44 *dd* (7.1, 1.6)	7.44 *dd* (1.5, 7.6)	7.37–7.33 *m*	7.36–7.34 *m*	7.36–7.34 m
6	7.47 *dd* (1.6, 8.2)	7.46 *m*	7.40–7.37 *m*	7.39–7.36 *m*	7.39–7.36 *m*
7	4.91 *d* (11.7)	4.94 *d* (11.7)	2.97 *t* (7.0)	2.97 t (7.0)	2.97 *t* (7.0)
	4.75 *d* (11.7)	4.75 *d* (11.7)			
8	−	−	3.91 *m*	3.91 *m*	3.91 *m*
			4.12 *dt* (6.2, 9.0)	4.13 *m*	4.13 *m*
					
1′	4.52 *d* (8.0)	4.52 *d* (7.9)	4.45 *d* (7.9)	4.45 *d* (8.0)	4.45 *d* (8.0)
2′	3.28 *m*	3.28 *m*	3.25 *d* (9.0)	3.23 *d* (8.0)	3.23 *d* (8.0)
3′	3.43 *m*	3.43 *m*	3.46 *d* (9.0)	3.45 *m*	3.45 *m*
4′	3.46 *d* (9.0)	3.46 *m*	3.41 *d* (9.4)	3.45 *m*	3.45 *m*
5′	3.53 *ddd* (2.0, 6.0, 9.0)	3.59 *m*	3.56 *dd* (2.0, 5.9)	3.39 *dd* (2.0, 9.0)	3.39 *dd* (2.0, 9.0)
6′	4.02 *dd* (2.0, 11.6) 3.73 *dd* (6.0, 11.6)	4.15 *dd* (1.9, 11.7), 3.85 *dd* (5.7, 11.7)	3.68 *dd* (5.8, 12.1) 4.03 *dd* (2.0, 12.1)	4.01 *dd* (1.9, 11.6), 3.70 *dd* (5.8, 11.5)	4.01 *dd* (1.9, 11.6), 3.70 *dd* (5.8, 11.5)
1″	5.11 *d* (3.2)	4.45 *d* (7.8)	5.05 *d* (1.4)	4.44 *d* (8.0)	5.09 *d* (3.2)
2″	4.01 *d* (3.2)	3.32 *m*	4.10 *dd* (1.5, 3.3)	3.30 *m*	3.98 *d* (3.2)
3″	−	3.42 *m*	3.92 *dd* (3.3, 5.7)	3.45 *m*	−
4″	4.06 *d* (10.1) 3.89 *d* (10.1)	3.59 *m*	4.05 *dd* (3.3, 5.9)	3.89 *m*	4.03 *d* (10.1) 3.87 *d* (10.1)
5″	3.66 *s*	3.30 *m*, 3.95 *dd* (5.4, 11.6)	3.80 *dd* (3.2, 12.2) 3.69 *dd* (5.0, 12.2)	3.89 *dd* (2.2, 12.3) 3.69 *dd* (5.0, 12.3)	3.63 *d* (1.7)
6″	−	−	−	−	−

Data collected in 80:20 D_2_O:CD_3_OD (4:1). Spectra were referenced to TSP-d_4_ at δ 0.00. Coupling constants in Hz are given in parentheses. Abbreviations: *s*—singlet; *d*—doublet; *t*—triplet; *dd*—doublet of doublets; *ddd*—doublet of double doublets; *m*—multiplet.

**Table 5:** NMR data of dihydro–rosarin (**12**) and dihydro–rosavin (**16**).

	(12)				(16)			
Position	δ_C_^a^	δ_H_^b^	COSY	HMBC	δ_C_^a^	δ_H_^b^	COSY	HMBC
1	145.3	−			145.3	−		−
2	131.6	7.30 *d* (7.0)	H-3/5, 4	C-4	131.6	7.31 *d* (7.1)	H-3/5, 4	C-3/5, 4, 7
3	131.5	7.35 *d* (7.5)	H-2/6, 4	C-1	131.5	7.35 *d* (7.5)	H-2/6, 4	C-1, 2/6
4	129.1	7.25 *t* (7.3)	H-2/6, 3/5	C-2/6	129.1	7.25 *t* (7.3)	H-2/6, 3/5	C-2/6
5	131.5	7.35 *d* (7.5)	H-2/6, 4	C-1	131.5	7.35 *d* (7.5)	H-2/6, 4	C-1, 2/6
6	131.6	7.30 *d* (7.0)	H-3/5, 4	C-4	131.6	7.31 *d* (7.1)	H-3/5, 4	C-3/5, 4, 7
7	34.3	2.71 *t* (7.6)	H-8	C-1, 2/6, 9	34.3	2.71 *t* (7.6)	H-8	C-1, 2/6, 8, 9
8	33.8	1.93 *dt* (6.7, 13.7)	H-7, 9	C-1, 9	33.8	1.93 *dt* (6.7, 13.7)	H-7, 9	C-1, 7, 9
9	72.8	3.88 *m*	H-8		72.8	3.89 *m*	H-8	
		3.65 *m*				3.65 *m*		
1′	105.5	4.41 *d* (7.9)	H-2′	C-9	105.9	4.42 *d* (7.4)	H-2′	C-9
2′	76.2	3.25 *d* (9.2)	H-1′, 3′	C-3′, 4′	76.2	3.28 *d* (9.0)	H-1′, 3′	C-1′, 3′, 4′
3′	78.9	3.45 *m*	H-2′	C-2′, 4′	79	3.45 *m*	H-2′	C-5′
4′	72.5	3.45 *m*	H-5′	C-3′	72.5	3.45 *m*	H-5′	C-5′
5′	72.7	3.41 *dd* (9.5, 2.5)	H4′	C-2′, 3′, 4′, 6′	72.7	3.39 *dd* (9.0, 3.5)	H4′	C-3′, 4′
6′	69.7	4.02 *dd* (11.5, 1.9)		C-4′, 5′	71.5	4.11 *dd* (1.9, 11.7)		C-1′
		3.68 *dd* (11.5, 6.0)				3.82 *dd* (5.5, 11.7)		
1″	111.2	5.04 *d* (1.3)		C-6′, 2″, C-3″	104.4	4.53 *d* (7.9)		C-2″
2″	87	4.02 *dd* (1.8, 3.6)		C-3″	72.1	3.58 *dd* (1.2, 5.4)		C-3″
3″	79.5	3.90 *dd* (6.2, 3.0)		C-4″	78.4	3.57 *d* (5.5)	H-4″	C-2″
4″	84	4.07 *dd* (1.6, 3.3)		C-3″	68.1	3.92 *d* (5.5)	H-3″, 5″	C-2″, 3″
5″	64.3	3.77 *dd* (12.3, 3.3)		C-3″	63.7	3.89 *dd* (12.3, 2.3)	H-4″	C-3″
		3.66 *d*d (12.3, 5.0)				3.71 *dd* (12.3, 5.8)		

Data collected in 80:20 D_2_O:CD_3_OD (4:1). Spectra were referenced to TSP-d_4_ at δ0.00. Coupling constants in Hz are given in parentheses. ^a^ Measured at 150 MHz; ^b^ Measured at 600 MHz; Abbreviations: *s*—singlet; *d*—doublet; *t*—triplet; *dd*—doublet of doublets; *dt*—doublet of triplets; *m*—multiplet.

## Supplementary Materials

The following are available online. Table S1: NMR chemical shift data of p-hydroxybenzylacetone-β-d-glucopyranoside (**5**); Table S2. NMR chemical shift data of chavicol-α-l-arabinofuranosyl-(1″→6′)-β-d-glucopyranoside (**18**) and chavicol-β-d-apiofuranosyl-(1″→6′)-β-d-glucopyranoside (**20**).
