# Supplementary material for: Phenylalkanoid Glycosides (Non-Salicinoids) from Wood Chips of Salix triandra × dasyclados Hybrid Willow
Source: Molecules. 2019 Mar 23;24(6):1152. doi: 10.3390/molecules24061152 (PMC6470679; doi:10.3390/molecules24061152)
Supplement: Supplementary file 1 [file molecules-24-01152-s001.pdf]

Supporting Information

Phenylalkanoid Glycosides (Non-Salicinoids) from Wood Chips of *Salix triandra* × *dasyclados* Hybrid Willow

Clarice Noleto-Dias, Yanqi Wu, Alice Bellisai, William Macalpine, Michael H. Beale, and Jane L. Ward

Contents

Table S1: NMR chemical shift data of *p*-hydroxybenzylacetone-β-D-glucopyranoside (5)

Table S2**.** NMR chemical shift data of chavicol-α-L-arabinofuranosyl-(1''→6')-β-D-glucopyranoside (**18**) and chavicol--β-D-apiofuranosyl-(1''→6')-β-D-glucopyranoside (**20**)

Table 1. NMR chemical shift data of *p*-hydroxybenzylacetone-β-D-glucopyranoside **(5).**

Pos[n]	δ_C[a]	δ_H[a]	$J_{H\text{-}H}$ (Hz); *multiplicity*[b]	COSY	HMBC	δ_C[c,d]	δ_H[c,d]	$J_{H\text{-}H}$ (Hz); *multiplicity*[b,c,d]
1	158.0	-	-	-	-	157.1	-	-
2	119.7	7.07	8.6; *d*	H-3	C-1, C-3/5, C-4	118.0	7.11	8.4; *d*
3	132.5	7.23	8.6; *d*	H-2	C-1, C-2/6, C-7	130.1	7.00	8.4; *d*
4	138.6	-	-	-	-	136.2	-	-
5	132.5	7.23	8.6; *d*	H-6	C-1, C-2/6, C-7	130.1	7.00	8.4; *d*
6	119.7	7.07	8.6; *d*	H-5	C-1, C-3/5, C-4	118.0	7.11	8.4; *d*
7	31.4	2.86	*m*	-	C-3/5, C-4, C-8, C-9	30.2	2.78	*m*
8	47.5	2.88	*m*	-	C-4, C-7, C-9	46.2	2.78	*m*
9	218.5	-	-	-	-	211.0	-	-
10	32.3	2.19	*s*	-	C-9	30.2	2.11	*s*
1′	103.4	5.05	7.7; *d*	H-2′	C-1	102.4	4.87	7.8; *d*
2′	75.9	3.53	7.8; *d*	H-1′	C-3′	75.1	3.39-4.40	*m*
3′	78.6	3.58	9.0; *d*	-	C-2′, C-4′	78.3	3.39-4.40	*m*
4′	72.6	3.47	9.0	-	C-5′	71.6	3.39-4.40	*m*
5′	78.9	3.43	*m*	H-6′	C-4′	78.2	3.39-4.40	*m*
6′	63.7	3.92	12.4, 2.1; *dd*	H-5′	C-5′	62.7	3.39-4.40	*m*
		3.73	*12.4, 5.8; dd*					

[a] Data collected in 80:20 D_2O:CD_3OD (4:1). Spectra were referenced to TSP-d4 at δ0.00.

[b] *s* singlet; *d* doublet; *dd* double doublet; *m* multiplet.

[c] Data collected in CD_3OD.

[d] Gao, L.; Xu, X.; Yang, J. Chemical constituents of the roots of *Rheum officinale*. *Chem. Nat. Compd.* **2013**, *49*, 603–605, doi:10.1007/s10600-013-0689-7.

Table S2: NMR chemical shift data of chavicol-α-L-arabinofuranosyl-(1''→6')-β-D-glucopyranoside **(18)** and chavicol--β-D-apiofuranosyl-(1''→6')-β-D-glucopyranoside **(20)**

	**18**					**20**				
Pos[n]	δ_C	δ_H	J_{H-H} (Hz); *multiplicity*[a]	COSY	HMBC	δ_C	δ_H	J_{H-H} (Hz); *multiplicity*[a]	COSY	HMBC
1	158.1	-	-	-	-	158.1	-	-		
2	119.9	7.07	8.7; *d*	H-3/5	C-1, C-4, C-3/5	119.8	7.07	8.6; *d*	H-3/5	C-1, C-4, C-3/5
3	132.9	7.22	8.7; *d*	H-2/6	C-1, C-2/6	132.8	7.22	8.6; *d*	H-2/6	C-1, C-2/6, C-7
4	138.5					138.4				
5	132.9	7.22	8.7; *d*	H-2/6	C-1, C-2/6	132.8	7.22	8.6; *d*	H-2/6	C-1, C-2/6, C-7
6	119.9	7.07	8.7; *d*	H-3/5	C-1, C-4, C-3/5	119.8	7.07	8.6; *d*	H-3/5	C-1, C-4, C-3/5
7	41.7	3.36	6.7; *d*	H-8		41.6	3.36	6.9; *d*	H-8	C-2/6, C-3/5, C-4, C-8
8	141.5	6.01	16.9, 10.1, 6.7; *ddt*	H-7, H-9		141.3	6.01	16.9, 10.1, 6.7; *ddt*	H-7, H-9	C-4, C-7
9	118.6	5.07	*m*	H-8		118.4	5.08	*m*	H-8	C7, C8
1′	103.5	5.04	7.7; *d*	H-2'	C-1, C-4'	103.5	5.03	7.6; *d*	H-2'	C-1, C-2'
2′	76.0	3.54	7.7; *d*	H-1'	C-1'	78.5	3.53	7.6; *d*	H-1', H-3'	C-1', C-3'
3′	72.7	3.50	9.1; *d*		C-1'	76.1	3.56	8.9; *d*	H-2'	C-4'
4′	78.6	3.57	9.0; *d*		C-3'	72.6	3.48	9.2; *d*		C-5', C-6'
5′	78.1	3.73	*m*			77.9	3.7	m	H-6'	
6′	69.8	4.04<br>3.70	11.1; *d*<br>*m*		C-3', C-1''	70.7	4.02<br>3.72	2.3, 11.4; *dd*<br>*m*	H-5'	C-4', C-5', C-1''
1″	111.2	5.00	1.3; *d*	H-2''	C-6', C-3'', C-4''	112.2	5.05	3.1; *d*	H-2''	C-6', C-2'', C-3'', C-4''
2″	84.2	4.06	1.5, 3.3; *dd*	H-1'', H-3''	C-1'', C-3''	79.6	3.94	3.1; *d*	H-1''	C-1'', C-5''
3″	79.7	3.89	3.3, 5.9; *dd*	H-2''	C-2'', C-4'', C-5''	82.3	-	-	-	-
4″	87.0	4.00	3.3; 5.9; *dd*		C-2'', C-3'', C-5''	76.6	3.83<br>3.99	10.1; *d*<br>10.1; *d*	H-4'', H-5''	C-1'', C-2'', C-3'', C-5''
5″	64.1	3.74<br>3.64	3.3, 12.3; *dd*<br>5.7, 12.3; *dd*		C-3'', C-4''	66.5	3.6	*s*	H-4''	C-2'', C-3'', C-4''

Data collected in 80:20 D_2O:CD_3OD (4:1). Spectra were referenced to TSP-d4 at δ0.00.

[a] *s* singlet; *d* doublet; *dd* double doublet; *m* multiplet.
